# Supplementary material for: Evaluation of Automatic Signal Detection of In Situ Hybridization for Detecting HPV DNA in Cervical Tissue Derived from Patients with Cervical Intraepithelial Neoplasia
Source: Cancers (Basel). 2024 Oct 15;16(20):3485. doi: 10.3390/cancers16203485 (PMC11506503; doi:10.3390/cancers16203485)
Supplement: Supplementary file 1 [file cancers-16-03485-s001.zip › cancers-3229864-supplementary.pdf]

Supplement

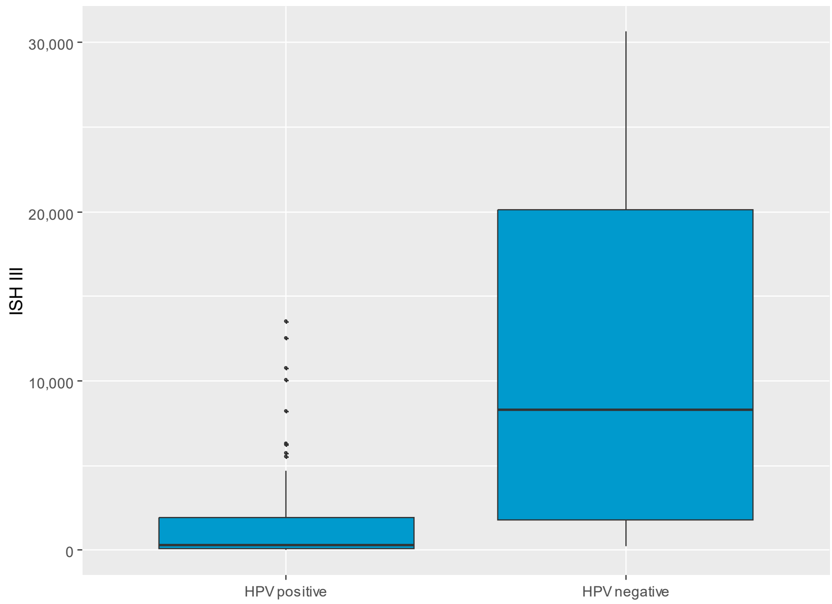

ISH- in situ hybridization

Figure S1. Boxplot chart visualizing level of ISH III in split to HPV positive and HPV negative groups

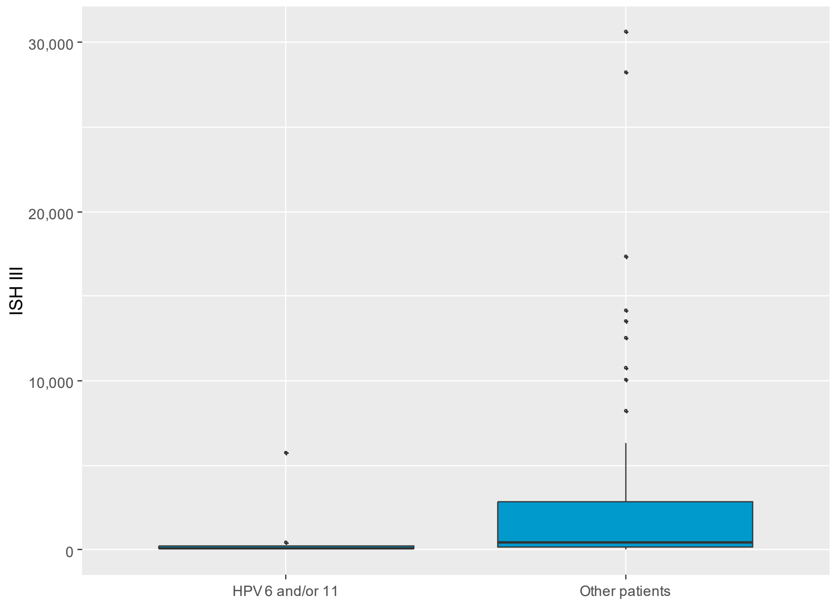

ISH- in situ hybridization

Figure S2. Boxplot chart visualizing level of ISH III in split to patients with HPV type 6 and/or 11 and other patients
